# Quantifying access block in a South African major trauma centre: a retrospective exploratory study

**DOI:** 10.1016/j.afjem.2026.100996

**Published:** 2026-07-24

**Authors:** Nicholas George Chapman, Ryoko Kinukawa, Elchanan Reuven Nudelman, Devorah Leah Wineberg

**Affiliations:** Trauma Directorate, Department of Surgery, Chris Hani Baragwanath Academic Hospital, Chris Hani Road, Diepkloof Zone 6, Soweto, Johannesburg, 1862, South Africa

**Keywords:** Trauma, Burns, Access block, Emergency medicine, South Africa, Interpersonal violence

## Abstract

**Introduction:**

Access block is defined as a delay in access to inpatient beds for patients from the Emergency Department (ED). It is a key contributor to ED overcrowding and is associated with worse patient outcomes. This study aimed to quantify access block within Chris Hani Baragwanath Academic Hospital’s Trauma Emergency Unit (TEU) and identify associated factors.

**Methods:**

A retrospective review was conducted of all consecutive patients presenting to the TEU resuscitation room from January 1 to December 31, 2022. Patients aged <14 years (<10 for burns) and those managed by other services were excluded. Data on demographics, clinical characteristics, resuscitation length-of-stay, interventions, and disposition were analysed. Access block was defined as a >8 h delay for the purposes of this study. Univariable analyses were performed to identify associations with access block.

**Results:**

During the study period, 2270 patients were admitted from the resuscitation room to the ward without first requiring the operating theatre, of whom 2091 had a recorded length-of-stay. Access block affected 61.3%. In unadjusted analysis, factors significantly associated with access block included a requirement for CT imaging (OR 4.17), intensive care unit (ICU) admission (OR 3.69), mechanical ventilation (OR 2.10), blunt mechanisms of injury (OR 2.19), presenting at night (OR 1.85), and an arrival shock index ≥1 (OR 1.32). Conversely, presenting with penetrating trauma (OR 0.61), burns (OR 0.50), and undergoing intercostal catheter insertion (OR 0.53) were associated with significantly lower odds of access block. Time-to-CT was significantly longer in patients with access block (366 vs 165 min; *P* < 0.01). Patients requiring mechanical ventilation had significantly longer length-of-stay (834 vs 631 min; *P* < 0.01), even once time-to-CT was accounted for.

**Conclusion:**

Most admitted patients were affected by access block. Associated factors included CT imaging, ICU admission, mechanical ventilation, and presentation at night. This corroborates the international consensus that whole-of-system solutions are necessary to remedy it.

**African relevance**

• Access block is a major contributor to emergency department overcrowding, and resource constraints may limit conventional solutions in low- and middle-income African settings.
• This study provides one of the first quantitative descriptions of access block within an African major trauma centre, enabling benchmarking for similar services.
• Most research into access block originates from high-resource settings; identifying associated factors in this setting may help inform context-appropriate strategies to improve patient flow and optimise use of limited resources.
• Our findings mirror the international consensus that access block and overcrowding are multi-disciplinary issues requiring multi-faceted solutions, and highlight priority areas for further investigation in comparable African settings.

## Introduction

“Access block” is a delay in accessing an inpatient bed for patients being admitted from the Emergency Department (ED), defined in high-income settings as greater than eight hours, and has been shown to be the single largest contributor to ED overcrowding [1,2]. It is a marker of imbalance between service supply and demand that has been shown to be associated with poorer quality of care. This manifests itself as increased rates of medical error, [3] poorer patient satisfaction [4] and higher patient mortality [[5], [6], [7], [8]].

The burden of trauma in South Africa is predominantly a result of interpersonal violence and road traffic collisions [9]. The incidence of injury has been shown to fluctuate significantly depending on the time of day and week, [10,11] and can also have periods of unpredictable surge, meaning that ED capacity must be sufficiently flexible to accommodate this.

Chris Hani Baragwanath Academic Hospital (CHBAH) is an urban major trauma centre and teaching hospital for the University of the Witwatersrand in Johannesburg, South Africa. It serves a population of approximately 3 million people locally as well as being a tertiary referral centre for the Gauteng province. The 3200-bed hospital is the largest in the southern hemisphere, and one of the largest in the world [12]. Inpatient admission capacity is limited to a 54-bed trauma ward, with overflow patients being admitted as outliers on other wards, or to the burns wards, if the patient presents exclusively with acute burns. Patients admitted are often subsequently transferred to outlier wards in order to improve bed capacity in the trauma ward. Patients with isolated pneumothoraces requiring an intercostal catheter (ICC) may be admitted directly from the resuscitation room to these outlier wards, if clinically appropriate following ICC insertion. Patients requiring mechanical ventilation or inotropic support can be admitted to one of eight intensive care unit (ICU) beds that are reserved for trauma patients, to one of four high-dependency beds in the trauma ward, or to one of six high-dependency beds in the burns unit.

Access block is a well-documented public health issue affecting acute care services worldwide, and there is now a modest body of research into it in a South African setting [[13], [14], [15], [16], [17], [18]]. Recent data from elsewhere in the Gauteng province demonstrated that ED lengths-of-stay may extend to several days [13]. We sought to quantify the extent of the problem within the CHBAH Trauma Emergency Unit’s (TEU) resuscitation room, and to identify risk factors that may contribute to designing a whole-of-system approach to remedying it.

## Methods

A retrospective document review was conducted using contemporaneous patient records manually entered by the TEU nursing staff into a patient register for all consecutive trauma patients assessed in the resuscitation room from January 1, 2022, to December 31, 2022. Trauma patients presenting to CHBAH TEU are streamed to the resuscitation room if meeting institutional criteria. These are based on vital signs at triage, mechanism and pattern of injury, and anticipated clinical course (detailed elsewhere by Chapman et al. [11]). Children aged <14 presenting with traumatic injury were excluded, as were children aged <10 presenting with burns. While these children are seen and treated in our resuscitation room, often with the assistance of TEU staff, these patients are managed by paediatric surgery. Patients presenting with acute surgical emergencies, managed by the Surgical Emergency Unit (SEU), were similarly excluded.

Data on baseline demographics, vital signs on arrival (oxygen saturation (SpO_2_); heart rate (HR); non-invasive blood pressure (NIBP) values such as systolic blood pressure (SBP); Glasgow coma scale (GCS)), mechanism and location of injury, procedures performed within the resuscitation room, time of arrival, time of departure, and patient disposition were extracted. From these, shock index (SI) and resuscitation length-of-stay (RLOS) were calculated, and percentage occupancy rates were modelled. The latter were approximated using a moving-window accumulation method based on hourly patient presentation data and RLOS data. Access block was defined as an RLOS >8 h (480 min). While this threshold is derived from general, and exclusively high-income setting ED populations, it was applied pragmatically in this study to allow comparison with existing literature [8,19].

A requirement for supplemental oxygen on arrival was defined as either an SpO_2_ <90% or being documented to have received supplemental oxygen at time of arrival. Tachycardia and bradycardia were defined as a HR >100/min and <60/min respectively, and hypotension was defined as an SBP <100 mmHg. Patient level of consciousness was at times described qualitatively in the nursing register. Where numerical GCS values were not recorded, qualitative descriptors documented in the nursing register were mapped to predefined GCS ranges. Those patients described as “alert” were assigned a GCS of 15, “confused” were assigned 13–14, “semi-conscious” were assigned 9–12, and “unconscious” were assigned ≤8. CT imaging was recorded as a binary variable indicating whether a CT scan was performed during the resuscitation room stay. Time-to-CT indicates the time elapsed until initial CT; repeat scans were not analysed separately.

Statistical analysis was performed using MedCalc version 23.2.1 (MedCalc Software Ltd., Ostend, Belgium). Univariable analyses were performed using the Pearson’s chi-squared test and Fisher’s exact test for categorical variables, Welch’s *t*-test for parametric continuous variables, and the Mann-Whitney U test for non-parametric continuous variables. Time-to-admission data were demonstrated using Kaplan-Meier curves. All tests of significance were two-tailed with an alpha of 0.05. Data completeness was assessed for all variables included in the analysis, though the pattern was not formally tested for randomness. The proportion of missing data was low (<5%) for all demographic and clinical variables; therefore, descriptive statistics were calculated using total group denominators. Time of arrival and departure exhibited greater missingness, with at least one of these two variables unavailable for 179 (7.9%) admitted patients, precluding RLOS calculation. Analyses of access block were performed using an available-case approach, with pairwise deletion from specific analyses where relevant data were unavailable. No imputation of missing data was performed.

**Table 1:** Baseline demographic and clinical characteristics of trauma patients presenting to the Trauma Emergency Unit (TEU) resuscitation room overall and by place of admission.

	Total (*n* = 5008)	Trauma (*n* = 2075)	Burns (*n* = 143)	ICU (*n* = 52)
**Demographics**				
Sex, male (%)	4412 (88.1%)	1837 (88.5%)	104 (72.7%)	43 (82.7%)
Age, years, median (IQR)	31.0 (25.0–39.0)	31.0 (25.0–39.0)	30.0 (23.0–40.0)	35.0 (29.8–39.0)
**Mechanism of injury**				
Stab (%)	1865 (37.2%)	840 (40.5%)	0 (0%)	3 (5.8%)
GSW (%)	931 (18.6%)	218 (10.5%)	0 (0%)	10 (19.2%)
MVC (%)	400 (8.0%)	150 (7.2%)	1 (0.7%)	15 (28.8%)
PVC (%)	394 (7.9%)	164 (7.9%)	0 (0%)	9 (17.3%)
MBC (%)	37 (0.7%)	11 (0.5%)	0 (0%)	0 (0%)
Fall (%)	225 (4.5%)	102 (4.9%)	0 (0%)	3 (5.8%)
Assault (%)	563 (11.2%)	315 (15.2%)	0 (0%)	4 (7.7%)
Mob assault (%)	286 (5.7%)	192 (9.3%)	2 (1.4%)	6 (11.5%)
Burns (%)	255 (5.1%)	65 (3.1%)	143 (100%)	0 (0%)
Other (%)	80 (1.6%)	63 (3.0%)	0 (0%)	2 (3.8%)
**Clinical condition on arrival**				
Pre-hospital intubation (%)	162 (3.2%)	42 (2.0%)	22 (15.4%)	10 (19.2%)
Supplemental O_2_ requirement (%)	239 (4.8%)	115 (5.5%)	4 (2.8%)	12 (23.1%)
HR >100/min (%)	1289 (25.7%)	531 (25.6%)	77 (53.8%)	30 (57.7%)
HR <60/min (%)	257 (5.1%)	90 (4.3%)	4 (2.8%)	3 (5.8%)
SBP <100 mmHg (%)	392 (7.8%)	121 (5.8%)	19 (13.3%)	14 (26.9%)
SI ≥1 (%)	504 (10.1%)	173 (8.3%)	36 (25.2%)	23 (44.2%)
GCS ≤8 (%)	464 (9.3%)	139 (6.7%)	33 (23.1%)	32 (61.5%)
**Procedure count (rate per 1000 patients)**				
ICC, count (rate)	695 (139)	531 (256)	1 (7.0)	17 (327)
CVC, count (rate)	259 (51.7)	88 (42.4)	26 (182)	14 (269)
ETT, count (rate)	467 (93.3)	174 (83.8)	19 (133)	22 (423)
RT, count (rate)	14 (2.8)	0 (0)	0 (0)	0 (0)
Surgical airway, count (rate)	4 (0.8)	0 (0)	0 (0)	0 (0)

Percentages are calculated using total group denominators. Some patients presented with multiple mechanisms of injury. Missing data for individual variables were low (<5%) and were not excluded from denominators. Procedure rate is per 1000 patients. Abbreviations: CVC, central venous catheter; ETT, endotracheal tube; GCS, Glasgow coma scale; GSW, gunshot wound; HR, heart rate; ICC, intercostal catheter; ICU, intensive care unit; IQR, interquartile range; MBC, motorbike collision; MVC, motor vehicle collision; PVC, pedestrian vehicle collision; RT, resuscitative thoracotomy; SBP, systolic blood pressure; SI, shock index.

This study received ethical approval from the University of the Witwatersrand Human Research Ethics Committee (M260235), and is reported in accordance with REporting of studies Conducted using Observational Routinely collected Data (RECORD) guidelines.

## Results

**Fig. 1:**
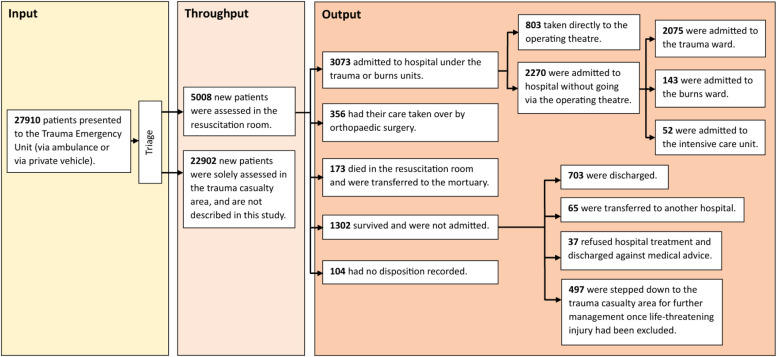
Patient flow through the Trauma Emergency Unit (TEU) resuscitation room.

During the study period, 5008 injured patients were assessed and treated in the TEU resuscitation room. Patient flow can be seen in Fig. 1, and baseline characteristics of those patients directly admitted can be seen in Table 1.

**Fig. 2:**
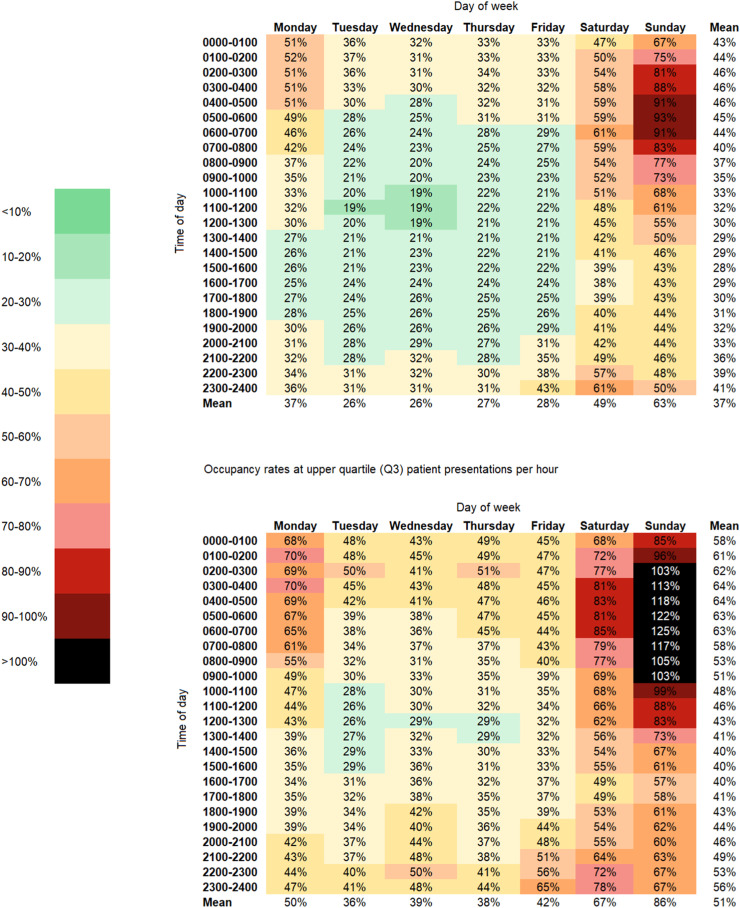
Heatmap matrix of modelled occupancy rates in the Trauma Emergency Unit's (TEU) 15-bed resuscitation room by day of week and time of day, assuming median (Q2) patient presentations per hour (top) and upper quartile (Q3) patient presentations per hour (bottom).

**Fig. 3:**
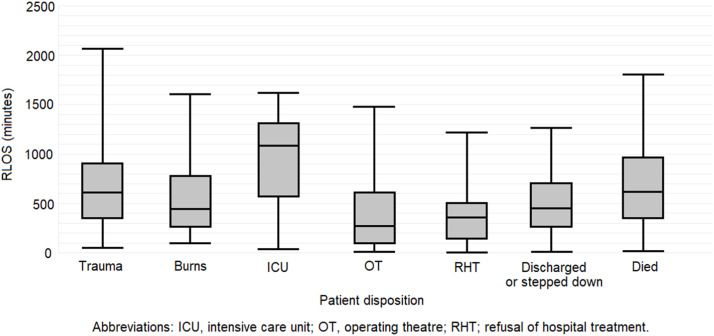
Box-and-whisker plots demonstrating resuscitation length of stay (RLOS, minutes) by patient outcome. Note that RLOS for those who died includes the duration of time before the deceased was taken to the mortuary, and is not the duration of survival in the resuscitation room.

Across all patients assessed in the TEU, mean RLOS was 572 min (95% CI 561–583). Median patient presentations per hour was 0.56 (IQR 0.45–0.70), but this varied significantly by hour of the day and day of the week. The effect of these factors on resuscitation room occupancy rates can be seen below (Fig. 2), as can the breakdown of RLOS by patient outcome (Fig. 3).

Of the 2270 patients admitted directly from the resuscitation room to the ward, 2091 (92.1%) had a time of arrival and departure recorded. Mean RLOS for these admitted patients was 657 min (95% CI 640–673), with access block affecting 1282 (61.3%) of them. Of those admitted to the trauma wards, 1182 (61.7%) were affected by access block, for the burns wards this number was 61 (47.7%), and for those admitted to the ICU this number was 39 (84.8%).

Factors associated with increased odds of access block were undergoing CT imaging (OR 4.17, 95% CI 3.31–5.25; *P* < 0.01), admission to the ICU (OR 3.69, 95% CI 1.60–8.66; *P* < 0.01), presentation with a blunt mechanism of injury (OR 2.19, 95% CI 1.81–2.65; *P* < 0.01), a requirement for mechanical ventilation (OR 2.10, 95% CI 1.50–2.95; *P* < 0.01), having an SI ≥1 on arrival (OR 1.32, 95% CI 1.07–1.63; *P* = 0.01), and presentation at night (2400–0759) compared to other times (OR 1.85, 95% CI 1.50–2.29; *P* < 0.01). Patients with access block also had a significantly longer time-to-CT (366 vs 165 min, *P* < 0.01).

Factors associated with lower odds of access block were presentation with burns (OR 0.50, 95% CI 0.37–0.67; *P* < 0.01), penetrating mechanisms of injury (OR 0.61, 95% CI 0.51–0.74; *P* < 0.01), ICC insertion (OR 0.53, 95% CI 0.43–0.65; *P* < 0.01), and presentation during the day (0800–1559) compared to other times (OR 0.81, 95% CI 0.67–0.97; *P* = 0.02).

Patients admitted to hospital following blunt mechanisms of injury had a mean RLOS of 744 min (95% CI 718–769). Even once time-to-CT was accounted for, patients with blunt trauma still had a longer RLOS than patients with other mechanisms of injury in whom CT imaging was performed, as mean post-CT RLOS was significantly longer (572 vs 465 min; *P* < 0.01).

Time-to-CT varied significantly by time of day. For patients presenting to the resuscitation room during the day (0800–1559) median time-to-CT was 247 min (IQR 150–405), for patients presenting during the evening (1600–2359) median time-to-CT was 227 min (IQR 139–376), and for those presenting overnight (2400–0759) median time-to-CT was 332 min (IQR 166–500), which was significantly longer than for both day and evening presentations (*P* < 0.01).

**Fig. 4:**
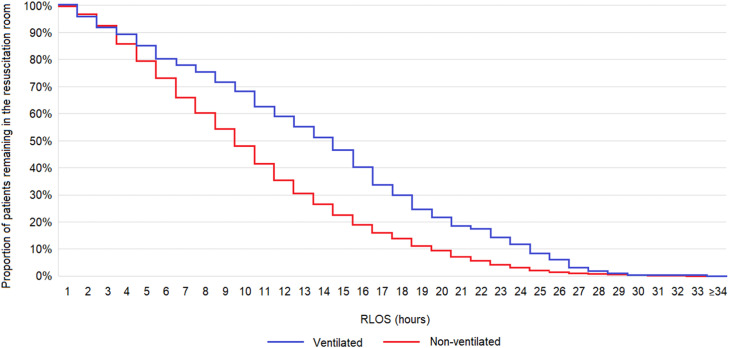
Kaplan-Meier curves demonstrating the proportion of admitted ventilated and non-ventilated patients remaining in the resuscitation room (%) by length-of-stay (hours).

A total of 264 (12.6%) admitted patients with a recorded RLOS required mechanical ventilation at some point during their time in the resuscitation room, 75.4% of whom experienced access block. Of these, 198 (75.0%) were admitted to the trauma ward, 37 (14.0%) were admitted to the burns ward, and 29 (10.9%) were admitted to the ICU. Mean RLOS for the ventilated group was 834 min (95% CI 780–887), while the mean RLOS for the non-ventilated group was significantly shorter at 631 min (95% CI 614–648) (*P* < 0.01). Kaplan-Meier curves demonstrating this separation can be seen below (Fig. 4). This occurred despite no significant difference in median SI between groups (*P* = 0.18). This association also persisted once time-to-CT was accounted for, as ventilated patients still had a longer mean post-CT RLOS than non-ventilated patients who also underwent CT imaging (703 vs 493 min; *P* < 0.01). This association only persisted for those ventilated patients admitted to the trauma wards, however (OR 2.87, 95% CI 2.01–4.11; *P* < 0.01), as there was no association between mechanical ventilation and access block for patients admitted to the burns wards (OR 0.73, 95% CI 0.34–1.57; *P* = 0.59) or the ICU (OR 0.60, 95% CI 0.10–3.23; *P* = 1.00).

## Discussion

Patients who arrive at hospital when rates of access block are >10% have been shown to have 10% higher mortality than their counterparts who present when there is access block to a lesser degree, [8] an association likely due to the effect of overcrowding on quality of care. Blunt mechanisms of injury were significantly associated with access block. This may reflect the greater complexity of this patient group, as blunt mechanisms such as motor vehicle collision (MVC) or pedestrian versus vehicle collision (PVC) are more commonly associated with multi-system injury and resource utilisation than penetrating trauma [20].

A requirement for CT imaging was significantly associated with access block, with time-to-CT being significantly greater overnight. At night, CT capacity is reduced, with just 1–2 CT scanners in operation, compared with 2–3 during the day. The exact number is contingent on the availability of radiographers. These must be shared with the remainder of the hospital, including the neighbouring Medical and Surgical Emergency Units (MEU and SEU). These factors may contribute to delays in both diagnosis and disposition and are consistent with the observed association between CT imaging and access block, as patients are not admitted to the ward until imaging has been performed.

Of patients managed in the resuscitation room, 12.6% required mechanical ventilation at some point during their stay. These patients had significantly longer RLOS than patients who did not require mechanical ventilation, which may reflect a combination of increased injury severity, ongoing stabilisation requirements, and limited availability of high-dependency beds. Likewise, admission to the ICU was associated with access block, irrespective of mechanical ventilation status. Prolonged RLOS for these patients may have important downstream implications for resuscitation room capacity, as it is equipped with just six bedside ventilators and one portable transport ventilator. Once at capacity, the TEU must notify the hospital superintendent and request that patients being transferred by emergency medical services are diverted elsewhere. This bypass period typically lasts four hours, and on average occurs 1–2 times per month. This is disruptive to service provision, may delay definitive care for those patients diverted, and still does not divert those arriving by private vehicle.

Resuscitation room occupancy demonstrates significant temporal variability, yet there can also be unpredictable periods of patient surge. This poses a unique challenge, as capacity must exist to accommodate the upper end of this range in patient volume, even at the expense of lower occupancy rates during weekdays, and its resourcing must be sufficiently flexible to accommodate these periods of surge. At current levels, >90% occupancy is being reached on Saturday nights even with median patient volumes. This means weekends with higher volumes are likely to see occupancy rates in excess of 100%. This is lower than the reported occupancy rates for some other South African EDs, [16] but these data pertain to only the resuscitation room, not the TEU more broadly. These data also do not take into consideration the fact that our resuscitation room is often utilised by other departments, all of which contributes to the demand for nursing staff, space, and equipment but which is not described in these data.

This study is limited by the incompleteness of these data. Of those patients admitted directly from the resuscitation room, 179 (7.9%) had no documented arrival or departure time. Missing data were low for most demographic and clinical data but may have introduced bias if not missing at random. The derivation of GCS from qualitative descriptors may introduce misclassification bias due to variability in interpretation. The proportion of cases requiring this approach could not be quantified, as mapping to pre-defined GCS ranges was performed at the time of data collection. This study is based on retrospective observational data and inferential analyses were limited to univariable comparisons which may be confounded by unmeasured factors such as injury severity, which was not described in these data. These limitations precluded meaningful multivariable modelling, and findings should therefore be interpreted as hypothesis generating. This study was conducted in a high-volume tertiary trauma centre with dedicated resuscitation facilities and specialist services, meaning the findings are most directly applicable to similar tertiary centres. Caution is required when extrapolating to smaller or non-specialist units, where patient mix, resource availability, and admission pathways differ. However, key themes identified in this study are likely relevant across a range of African ED settings, albeit to different degrees.

It should also be noted that RLOS is a unidimensional performance indicator that assumes that the length-of-stay metric is negatively correlated with overall quality of care. The definition of access block used in this study is derived from general ED populations in high-income countries and may not fully reflect the unique operational context of a trauma resuscitation environment in a lower-resource setting, where patients often require prolonged stabilisation and complex decision-making prior to admission. As such, some degree of extended RLOS may be clinically appropriate and not solely indicative of system inefficiency. However, while this may affect external validity, the use of a standardised threshold for access block allows comparison with existing literature and provides a pragmatic benchmark for evaluating patient flow within this setting. The TEU’s out-of-hours staffing model also means that patients admitted to the ward are still the responsibility of resuscitation room medical staff, a system unlike that of a typical ED. It is unclear whether the increased risk to patients ascribed to access block in the international medical literature applies in this context. For selected patients, staying in the resuscitation room may be preferable over transfer to a ward where medical oversight is poorer, but this may occur at the expense of resuscitation room overcrowding and further overburdening already limited resources.

The findings of this study mirror the international consensus that overcrowding and access block are multi-disciplinary issues requiring multi-faceted solutions. Notably, the downstream factors potentially implicated involve services over which the unit has no control. This supports the argument that durable solutions to overcrowding require whole-of-system accountability, not individual department-level intervention [21]. In resource-limited settings, expansion of inpatient bed numbers and staff may not be feasible, and given that overcrowding has a predictable temporal pattern, pragmatic operational interventions may be preferable to reduce RLOS. These may include improvements in workflow coordination between services, alterations in CT availability to reflect periods of peak demand and therefore improve throughput, and optimisation of existing inpatient capacity through bed managerial staff to improve output.

## Conclusion

This retrospective study demonstrates that access block is a pervasive issue, affecting 61.3% of patients admitted directly from the resuscitation room. In unadjusted analysis, access block was associated with a requirement for CT imaging, mechanical ventilation, admission to the ICU, and presentation at night, though causality cannot be inferred. Notably, several of the implicated factors relate to services beyond the unit’s direct control, consistent with international evidence that overcrowding and access block are whole-of-system problems rather than those any single department can resolve alone. Durable improvement is therefore likely to depend on hospital-wide ownership and active coordination between services, rather than on infrastructure expansion alone, which is frequently infeasible in resource-limited settings. Pragmatic operational measures such as improved inter-departmental workflow, CT availability aligned to peak demand, and active bed management represent achievable short-term levers whose utility should be confirmed through further interventional study.

## Dissemination of results statement

Findings from this study were shared with the clinical leadership of the Chris Hani Baragwanath Academic Hospital Trauma Directorate to inform ongoing service planning and quality improvement initiatives.

## Funding

No funding body was involved in the study design, data analysis, results interpretation, or writing of the manuscript.

## CRediT authorship contribution statement

**Nicholas George Chapman:** Conceptualization, Methodology, Investigation, Data curation, Formal analysis, Writing – original draft, Writing – review & editing. **Ryoko Kinukawa:** Data curation, Writing – review & editing. **Elchanan Reuven Nudelman:** Investigation, Writing – review & editing. **Devorah Leah Wineberg:** Conceptualization, Methodology, Supervision, Writing – review & editing.

## Declaration of competing interest

The authors declare that they have no known competing financial interests or personal relationships that could have appeared to influence the work reported in this paper.

## Data Availability

The data for this study were obtained from institutional medical records in the Chris Hani Baragwanath Academic Hospital’s (CHBAH) Trauma Emergency Unit (TEU) in 2022. Researchers wishing to access the TEU 2022 dataset must apply through the Trauma Directorate. Anonymised summary data for this article are available from the corresponding author on reasonable request, subject to institutional approval.

## References

[1] Australasian College for Emergency Medicine (2022). Policy on standard terminology P02 V9 [internet]. https://policy.acem.org.au/index.php/policies-menu/p02-policy-on-standard-terminology.

[2] Australasian College for Emergency Medicine (2022). Access block position statement S127 [internet]. https://acem.org.au/getmedia/c0bf8984-56f3-4b78-8849-442feaca8ca6/S127_v01_Statement_Access_Block_Mar_14.aspx.

[3] Kolikof J., Shaw D., Stenson B., Balaji L., Grossestreuer A., Chiu D. (2025). Emergency department boarding, crowding, and error. J Am Coll Emerg Physicians Open.

[4] Viccellio P., Zito J.A., Sayage V., Chohan J., Garra G., Santora C. (2013). Patients overwhelmingly prefer inpatient boarding to emergency department boarding. J Emerg Med.

[5] Cheng T., Peng Q., Jin Y., Yu H., Zhong P., Gu W. (2022). Access block and prolonged length of stay in the emergency department are associated with higher patient mortality rate. World J Emerg Med.

[6] Cha W.C., Cho J.S., Shin S.D., Lee E.J., Ro Y.S (2015). The impact of prolonged boarding of successfully resuscitated out-of-hospital cardiac arrest patients on survival-to-discharge rates. Resuscitation.

[7] Singer A.J., Thode H.C., Viccellio P., Pines J.M (2011). The association between length of emergency department boarding and mortality. Acad Emerg Med.

[8] Jones P.G., van der Werf B. (2021). Emergency department crowding and mortality for patients presenting to emergency departments in New Zealand. Emerg Med Australas.

[9] Prinsloo M., Mhlongo S., Roomaney R.A., Maineau L., Mamashela T.A., Dekel B. (2024). Injury mortality in South Africa: a 2009 and 2017 comparison to track progress to meeting sustainable development goal targets. Glob Health Action.

[10] Chapman NG, Kinukawa R, Nudelman ER, Wineberg DL (2026). The contemporary burden of major trauma in Soweto: a descriptive study from the resuscitation room of Chris Hani Baragwanath academic hospital. S Afr J Surg.

[11] Bhana M., Fru P., Plani F. (2022). A long walk to freedom: the epidemiology of penetrating trauma in South Africa – analysis of 4697 patients over a six-year period at Chris Hani Baragwanath academic hospital. S Afr J Surg.

[12] Botchway M.T., Kruger D., Manful C.A., Grieve A. (2021). The scope of operative general paediatric surgical diseases in South Africa – the Chris Hani Baragwanath experience. Ann Pediatr Surg.

[13] Motimele L., Lalloo V., Sefala T., Engelbrecht A., Majake-Mogoba L., Basu D. (2025). Factors contributing to extended length of stay in the emergency department and potential strategies for improving patient flow in a central hospital in the Gauteng Province, South Africa. Afr J Emerg Med.

[14] Mashao K., Heyns T., White Z. (2021). Areas of delay related to prolonged length of stay in an emergency department of an academic hospital in South Africa. Afr J Emerg Med.

[15] Hendrickse C.A., Hodkinson P., van Hoving D.J (2023). An initiative to reduce psychiatric boarding in a Cape Town emergency department. S Afr J Psychiatr.

[16] Ahiable E., Lahri S., Bruijns S. (2017). Describing the categories of people that contribute to an emergency centre crowd at Khayelitsha hospital, Western Cape, South Africa. Afr J Emerg Med.

[17] De Vries E., Raubenheimer P., Kies B., Burch V.C (2011). Acute hospitalisation needs of adults admitted to public facilities in the Cape Town Metro district. S Afr Med J.

[18] Schoeman D.H (2020).

[19] Richardson D.W., Mountain D. (2009). Myths versus facts in emergency department overcrowding and hospital access block. Med J Aust.

[20] Fitch J.L., Albini P.T., Patel A.Y. (2019). Blunt versus penetrating trauma: is there a resource intensity discrepancy?. Am J Surg.

[21] Morley C., Unwin M., Peterson G.M., Stankovich J., Kinsman L. (2018). Emergency department crowding: a systematic review of causes, consequences and solutions. PLoS One.

